# How Daily Obstacles Affect Frontline Healthcare Professionals’ Mental Health during Omicron: A Daily Diary Study of Handwashing Behavior

**DOI:** 10.3390/ijerph19148748

**Published:** 2022-07-18

**Authors:** Nazeer Hussain Khan, Sajid Hassan, Sher Bahader, Sidra Fatima, Syed Muhammad Imran Haider Zaidi, Razia Virk, Kexin Jiang, Enshe Jiang

**Affiliations:** 1 School of Life Sciences, Henan University, Kaifeng 475004, China; kakakhan3514@gmail.com; 2 Department of Psychology, International Islamic University, Islamabad 44000, Pakistan; sajid.mscp397@iiu.edu.pk (S.H.); sher.mscp420@iiu.edu.pk (S.B.); 3 University Gillani Law College, Bahauddin Zakariya University, Multan 60000, Pakistan; sidrafatima.615@gmail.com; 4 Department of Applied Psychology, Government College University Faisalabad, Faisalabad 38000, Pakistan; imranhaider@gcuf.edu.pk; 5 Department of Bio-Sciences, University Wah, Rawalpindi 47040, Pakistan; razia.virk@uow.edu.pk; 6 Institute of Nursing and Health, Henan University, Kaifeng 475004, China; abcjkexin@126.com

**Keywords:** COVID-19 phobia, burnout, work-related stress, mental health, handwashing behavior, omicron wave

## Abstract

Based on coping theory, the current research examines how and why COVID-19 phobia affects frontline healthcare professionals’ mental health, as well as their burnout and work-related stress. We focused on the mediating role of burnout and work-related stress in this study. In the current study, we also examined the moderating influence of healthcare professionals’ handwashing behavior using the Hayes Process model. We employed a daily diary approach to collect data from respondents in Pakistan’s frontline healthcare professionals (*n* = 79, 79 × 10 = 790) who were directly treating COVID-19 patients during the omicron wave. According to the findings of the study, COVID-19 phobia significantly disturbs healthcare professionals’ mental health, as well as significantly strengthens burnout and work-related stress. The findings also demonstrated that burnout significantly negatively influences mental health. The mediation influence of burnout and work-related stress in the association between COVID-19 phobia and mental health has shown to be significant. The moderation analysis revealed that high handwashing behavior significantly buffers the negative impact of COVID-19 phobia, as well as the adverse effect of burnout on healthcare professionals’ mental health. Moreover, our findings have theoretical and managerial implications, as well as new research directions for scholars to understand the adverse impact of daily obstacles on professionals’ (nurses and doctors, etc.) mental health and work performance, as well as issues based on resource conversation philosophy.

## 1. Introduction

Owing to its multiple mutations, immune evasion, and fast transmissibility, in the COVID-19 pandemic, the SARS-coronavirus-2 (SARC-CoV-2) omicron variant emerged as a very alarming wave of infection. According to recent statistics, SARC-CoV-2 has infected over 421 million people globally, with over 5.8 million deaths. In November 2021, a new SARS-CoV-2 variant of concern (VOC) named “omicron” was first detected in South Africa [1]. Although the omicron variant is less severe than earlier variants of SARC-CoV-2, the rapid transmissibility of this variant has imposed severe effects on other health conditions and led to overwhelmed health systems across the globe. Furthermore, the risks of hospitalization, intensive care unit admission, and mortality from omicron are still considerable in non-vaccinated people [2].

**Figure 1:**
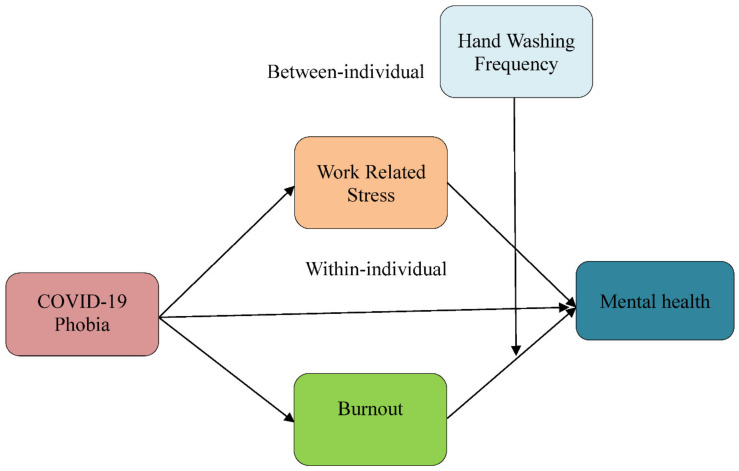
Research model.

As frontline respondents to this deadly pandemic, frontline healthcare workers (F-HCWs), including doctors, nurses, and paramedics, are most prone to contracting this fatal infection. F-HCWs are working as key players to mitigate the effect of the COVID-19 pandemic and its consequences, as well as implementing preventive measures to stop the transmission of the virus. Going through the published literature, it has been well investigated that, regarding COVID-19, these F-HCWs have significant psychological stress, worry, and anxiety and have been struggling with uncertainty since the beginning of the COVID-19 pandemic [2]. Similarly, in the context of the omicron upsurge, difficult tolerance of preventive obstacles and high uncertainty outcomes have been observed and found to correlate with stress and anxiety in the daily life of frontline healthcare professionals’ mental health. Thus, it is crucial to explore this correlation between F-HCW’s mental health and ongoing pandemic uncertainty and obstacles in daily life. In the present study, considering the clinical and psychological importance of association, by employing coping theory, we investigated and predicted the linkages between daily obstacles and frontline healthcare professionals’ mental health during omicron under the five essential components: cognitive efforts, behavioral efforts, internal demands, external pressures, and resources (Figure 1).

The coping theory was used in this study to investigate the predicted linkages. According to the definition of coping, it is “the cognitive and behavioral efforts made to deal with specific external and/or internal demands that are regarded as demanding or exceeding the person’s resources” [3]. Coping is concerned with a person’s adaptive actions in response to stressful events in his or her life. Coping theory is the most extensively used and accepted in psychology under the contextual model [4]. The five essential components of this idea are cognitive efforts, behavioral efforts, internal demands, external pressures, and resources [5].

The things that individuals deal with, as well as their significance and relevance, are evaluated (primary appraisal). Similarly, during the omicron wave, medical experts ask themselves, “What am I risking in this position?” The major issue is assessing the likely implications of this occurrence (specific internal/external demands), as well as the disruption’s relevance. In monitoring, disruptive events are divided into two categories: challenges and threats. Individuals will obtain information regarding COVID-19 or omicron in this study. Challenges are events that are believed to have good results. In contrast, threats are events that have negative effects (they will receive stress regarding COVID-19 and are prone to threat, psychological distress, and adverse work outcomes). In addition to appraising the severity of an experience, people frequently examine the coping skills (as an internal resource) available to them (secondary appraisal). People decide how much control they have over the problem and what they should do about it using the coping abilities at their disposal [4]. As previously stated, the coping theory is suited for analyzing the hypothesized model since it encompasses the complete mechanism of how health workers would react to COVID-19 while performing their duties, specifically in the omicron wave.

## 2. Theory and Hypotheses Development

### 2.1. Coping Theory

The coping theory was used in this study to investigate the predicted linkages. According to the definition of coping, it is “the cognitive and behavioral efforts made to deal with specific external and/or internal demands that are regarded as demanding or exceeding the person’s resources” [3]. Coping is concerned with a person’s adaptive actions in response to stressful events in his or her life. Coping theory is the most extensively used and accepted in positive psychology and cognitive psychology under the contextual model [4,6,7]. Coping theory serves two primary functions. One is in control of the worry or emotions caused by the unpleasant situation (emotion-focused coping). The alternative is to quickly change the components of the stressful situation in order to deal with the problem that is causing the stress [3]. The five essential components of this idea are cognitive efforts, behavioral efforts, internal demands, external pressures, and resources [5]. Behavioral efforts, such as gathering more information and proof and confronting persons, aim to change the circumstance itself, whereas cognitive efforts, such as acceptance, distance, and escape attempts (mental health goal in this study), attempt to change the context of the case [3]. Individual expectations or aims are to be satisfied by the environment, such as an individual’s desire to attain a hard job (work-related stress in this case) despite the obstacles associated with efficiently doing a particular sort of work. External demands originate from the situational or social environment and must be addressed by individuals. Finally, the resources available to individuals (monetary, material, physiological, physical, psychological, and behavioral) influence how they cope [3,5].

Individuals’ ability to deal with circumstances, as well as their significance and relevance, are assessed (primary appraisal). Similarly, during the omicron wave, medical experts will ask themselves, “What am I risking in this position?” The major issue is assessing the likely implications of this occurrence (specific internal/external demands), as well as the disruption’s relevance. In monitoring, disruptive events are divided into two categories: challenges and threats. Individuals will obtain information regarding COVID-19 or omicron in this study. Challenges are events that are believed to have good results, whereas threats are events that are seen to have negative effects (they will receive stress about COVID-19 and are prone to threat, psychological distress, and adverse work outcomes). In addition to appraising the severity of an experience, people frequently examine the coping skills (as an internal resource) available to them (secondary appraisal). People decide how much control they have over the problem and what they should do about it using the coping abilities at their disposal [4]. As previously stated, the coping theory is suited for analyzing the hypothesized model since it encompasses the complete mechanism of how health workers would react to COVID-19 while performing their duties, specifically in the omicron wave.

### 2.2. COVID-19 Phobia, Burnout, Work-Related Stress, and Mental Health

According to a large-scale study, the entire Iranian population is terrified of the COVID-19 disease [8]. Prior studies have shown that specific phobias can have a negative impact on an individual’s mental health [9,10,11], as well as a negative impact on individual happiness [12,13]. Phobia also refers to a disproportional fear reaction to anxiety or a fear-provoking object or situation [14]. Data on COVID-19 phobias are needed by healthcare practitioners so that governments may create COVID-19 transmission control measures that do not cause psychological anguish [15]. The World Health Organization is concentrating its resources and efforts on the pandemic and has established national strategies to combat the virus’s spread, such as social distance measures, homestay guidelines, online education, and government laws [16,17].

Although these health policies and practices may successfully restrict COVID-19 growth, their adoption is extremely likely to create COVID-19-related phobia or dread among healthcare workers, resulting in psychological trauma and mental health flows, such as stressors and mental problems [18,19,20]. It suggests that face-to-face contact with COVID-19 patients and disturbing health professionals’ daily living routines may have heightened fear of infection and mental health problems. Researchers have invented coronavirus phobia to refer to excessive fear due to the COVID-19 pandemic [21,22]. It has been demonstrated that various individuals have COVID-19-related phobia and are concerned about their future due to a perceived lack of control and vaccination. Because of the pandemic, uncertainty, high mortality rate, and economic decline, people are more likely to develop corona-phobic reactions [23]. In this scenario, findings of Nagy revealed that a specific phobia (COVID-19) could significantly influence burnout and other mental health symptoms [24]. Furthermore, another study claimed that dealing with COVID-19′s patients causes a variety of stressors that disturb healthcare professionals [25]. For example, in Wuhan, COVID-19 frontline workers to physicians and nurses who were not frontline workers and were functioning as normal and discovered that COVID-19 frontline workers had a much greater risk of burnout and death phobia than other participants [26]. Similarly, death anxiety is the main cause of phobia [27]. Moreover, based on the current situation and literature, we can say that COVID-19 phobia in health professionals may disturb their mental health. As a result, in this study, the following hypothesis is suggested.

**Hypothesis** **1** **(H1).** *COVID-19 phobia has a negative impact on health professionals’ mental health*.

Burnout is a state of emotional, physical, and mental exhaustion brought on by continuous and severe stress. It occurs when you are emotionally exhausted, overwhelmed, and unable to satisfy incessant expectations [28]. Healthcare workers who cannot leave the hospital for an extended period have no choice but to shift their attention to COVID-19 disease [29]. A study found that, during treating COVID-19 patients, healthcare professionals (HCPs) experience psychological stressors, emotional, and professional problems [30], and these experiences, such as psychological stressors, may raise burnout levels among health professionals. In contrast, scholars have documented that burnout is linked to increased phobia [31].

According to a study [32], particular situational phobia indirectly increases the risk of burnout through the stressor. Traumatic stress is the consequence of psychologically stressful situations, and psychologically stressful situations become a cause of high burnout [33]. It is also claimed that, during a traumatic event, when a person is frustrated and experiencing burnout from a situation, he or she would avoid these situations and, as a consequence, will decide to leave such settings and all situational dread (COVID-19) [34,35]. Overall, few studies have directly and indirectly examined the link between COVID-19 phobia and burnout. For example, burnout is significantly linked with prolonged stress and psychiatric symptoms (death anxiety and fear of situational) [36]. However, on the basis of the previous finding, we can assume that COVID-19 phobia may increase burnout in a healthcare worker.

**Hypothesis** **2** **(H2).** *COVID-19 phobia has a positive impact on healthcare professionals’ burnout*.

Burnout is emotional fatigue and a loss of emotional resources caused by excessive levels of stress at work [37,38], while the multidimensional theory of burnout suggests that burnout is a state that arises as a result of a long-term misalignment or error between a person’s workload, level of control, and adequate reward for the job in health care nursing [39]. Moreover, according to Maslach, workload, sufficient control, and a lack of adequate payment for the job are elements in burnout, leading to mental health problems among healthcare nurses [40,41].

Researchers found that burnout causes unfavorable changes in nurses’ attitudes and behaviors, which are often connected to workers’ dissatisfaction with the ideals [42,43]. It was also revealed that nurses’ mental health is harmed by such negative attitudes and behaviors [44]. Burnout has been shown to have a major negative impact on mental health in several types of research. A study from Wuhan, for example, compared oncologists and oncology nurses who were COVID-19 frontline workers to physicians and nurses who were not frontline workers and working as usual and found that COVID-19 frontline workers had a significantly higher rate of burnout and mental health problems than the other participants [45]. We can also assume that burnout may increase mental health problems based on theory and literature. As a result, we propose the following hypothesis.

**Hypothesis** **3** **(H3).** *Burnout has a negative impact on the mental health of health professionals*.

### 2.3. Mediating Role of Burnout and Work-Related Stress

In the context of mental health performance, burnout is considered a relevant construct that belongs to health professionals’ performance and mental health [46]. It has been found that burnout mediates the link between specific phobia and mental illness [47]. Moreover, studies concluded that burnout significantly affects the relationship between specific phobia and mental illness [48,49]. In this regard, the current study shows that burnout may play a moderating role in the link between COVID-19 phobia and mental illness for the following reasons. First, burnout is a negative attitude that interferes with positive motivation and inspiration. Second, burnout has been linked to a wide range of unfavorable situations (COVID-19 pandemic) that influence many sorts of workers, their organizations, their performance, as well as mental health [50]. In this setting, this study assumes that healthcare professionals’ burnout may intervene to establish a link between COVID-19 phobia and mental health. As a result, the following hypothesis can be put forth.

**Hypothesis** **4** **(H4).** *Burnout significantly mediates the relationship between COVID-19 phobia and mental health*.

Work-related stress is the response that people experience when they are confronted with work demands and pressures that are not matched to their knowledge and abilities in a specific circumstance (pandemic) and that put their ability to cope under strain [51,52,53]. The study reported that work-related stress is a rising concern worldwide, affecting not just employees’ mental health and well-being but also individual productivity [54]. It has been observed that this pandemic (COVID-19) has left us with feelings of uneasiness, fear, and instability, among other things, and the pressure in the workplace is greater than ever [55,56]. The effects of the COVID-19 pandemic and the workload on health professionals were examined [57]. Workload pressure, task dependency, professional isolation, and familial involvement in work, job uncertainty, fear of infection, financial loss, stigma, and social exclusion are all major stressors for working people [58,59,60], and these stressors may strengthen psychological distress. Shortly, previous excellent literature has revealed that work-related stress might enhance COVID-19 phobia, as well as psychological distress in healthcare practitioners, both directly and indirectly. Another study suggested that Work-related stress (WRS) is a stressful experience made worse by an employee’s employment [61]. Several scholars agree that work-related stress negatively influences nurses’ physical and mental health [62,63]. However, scholars argue that stress results when that pressure becomes overwhelming or otherwise uncontrollable, which may lead to psychological distress. According to cognitive-mediational theory, our emotions are determined by our appraisal of the stimulus. Stress is also a special relationship between a person and the environment that a person views as exceeding their resources and endangering their mental health [3]. This study proposes the following hypothesis to anticipate the mediating influence of work-related stress.

**Hypothesis** **5** **(H5).** *Work-related stress significantly mediates the relationship between COVID-19 phobia and mental health*.

### 2.4. Handwashing as a Moderator

The transactional model of stress [3] elucidates components that may aid in the decrease in dread feelings associated with COVID-19. As previously stated, emotion suppression is an emotion-focused coping approach that can help limit the impact of the perceived risk. Individuals can utilize problem-focused coping mechanisms or coping theory to lessen the influence of COVID-19-related phobia on emotion suppression while simultaneously lowering the impact of COVID-19-related fear on emotion suppression [3]. To put it another way, problem-focused coping entails strategies for enduring and reducing the threat, whereas suppression is concerned with minimizing one’s emotional response to the danger. This follows prior stress theories, which propose that problem-focused coping can help mitigate the harmful effects of stressors when faced with situational demands [64]. As a result, coping theory and problem-solving skills are expected to mitigate the negative effects of the stressor (in the context of COVID-19 fear). The relationship between stress and psychological well-being is moderated by emotion-focused coping, whereas problem-focused coping has been shown to moderate it [65,66]. Handwashing is one of the most important problem-focused coping methods during the current omicron wave. Indeed, the World Health Organization’s (WHO) COVID-19 website’s first item of advice is to “clean your hands routinely and thoroughly with an alcohol-based hand rub or wash them with hot water and soap” [67]. Given that regular handwashing destroys the virus [68], it acts as a safeguard against the COVID-19 threat [68]. This is important since phobias or fears of something frequently lead to a loss of control, which may be addressed by participating in proactive coping methods, such as handwashing, intended to reduce COVID-19 fear [69,70,71]. While the effect of handwashing on the association between COVID-19-related phobia and mental health has not been scientifically examined, there is evidence that handwashing lessens the impact of stressors.

Handwashing, for example, has been demonstrated to alleviate the negative effects of personal threats by evoking positive sentiments and decreasing cognitive interference [72,73]. We hypothesize that regular handwashing may moderate the impact of COVID-19-related phobia based on the accompanying explanations:

**Hypothesis** **6** **(H6).** *Handwashing moderates the negative relationship between burnout and mental health such that the negative association is weak when the handwashing frequency is great*.

**Hypothesis** **7** **(H7).** *Handwashing moderates the negative relationship between COVID-19 phobia and mental health such that the negative association is weak when the handwashing frequency is great*.

**Hypothesis** **8** **(H8).** *Handwashing moderates the negative relationship between work-related stress and mental health such that the negative relationship is weak when the handwashing frequency is great*.

## 3. Method

### 3.1. Sampling and Data Collection Procedures

Sample and procedure full-time health professionals were recruited physically (where researchers are providing services as a psychologist), as well as through electronic postings on several representative social media sites (e.g., Facebook, LinkedIn, WhatsApp). The recruitment advertisements explained the process of the study, inclusion criteria, and compensation for participation (a $5 gift card for each participant). The study was completed in two stages. In Stage 1, participants were emailed their consent details, and, in stage 2, participants completed daily surveys over two weeks (ten consecutive working days). 

Sample and procedure full-time health professionals were recruited both physically (where 2st and 3nd authors provide services as a psychologist) and electronically (via posts on various popular social networking sites) (e.g., Facebook, LinkedIn, WhatsApp). The recruiting ads outlined the study’s procedure, inclusion criteria, and pay ($5 gift card for each participant). 

The research was split into two parts. Participants were emailed their consent details in stage 1, and they completed daily surveys over two weeks in stage 2 (ten consecutive working days). To be eligible, participants had to be full-time (more than 30 h per week) health workers aged 18 to 65 who were proficient in English. During the research period, participants must work during typical working hours (e.g., 9 a.m. to 5 p.m., Monday through Friday) and have access to email and the internet at home and work. 

One-hundred-ninety-three people expressed interest in the study by contacting the researchers and sharing their email addresses. Eligible participants were emailed a link to a detailed research overview, an informed consent form, and a one-time first survey. After individuals who completed the consent form at the first stage and whose initial consent was returned, Stage 2 of the research began. For 10 consecutive workdays, participants were emailed a diary survey after work between 5:30 and 9:00 p.m. (i.e., over two weeks). As is common in daily diary research (see Table 1), 103 participants missed some of the daily surveys (*n* = 29), who did not submit their survey questionnaire, *n* = 46 who did not show any response after 4 days, *n* = 26 who did not submit their responses after one week study, and 11 who did not show responses to some items). More than 40 percent of the 10 diary questionnaires were completed by all participants (1 survey in a day for 10 days as seen in Table 1). As a result, our final acceptable sample consists of *n* = 79 (79 × 10 = 790) people having day-level data see Table 2. The participants’ ages vary from 21 to 65, with an average age of 35.41 (SD = 7.62) and an average of 8.45 (SD = 94) daily hours worked. The sample’s details and additional descriptions are listed in Table 2.

### 3.2. Measurement Scale

The measurement scales used in this study were similar to those used in previous studies. The participants’ responses were collected using four valid and reliable questionnaires: COVID-19 phobia scale, work stress questionnaire, positive mental health questionnaire, burnout questionnaire, and handwashing behavior.

#### 3.2.1. COVID-19 Phobia Scale

The COVID-19 phobia scale (C19P-S) is a 20-item scale that was developed to assess COVID-19 phobia in the general public. Answers are provided on a five-point Likert scale, with 1 representing “strongly disagree,” 2 representing “disagree,” 3 representing “neither agree nor disagree,” 4 representing “agree,” and 5 representing “strongly agree.” The final score is obtained by combining the scores for all 20 items; hence, the total score range is 20–100. Higher scores indicate a higher level of COVID-19 phobia. For this analysis, Cronbach’s alpha value was 0.91 [22].

#### 3.2.2. Work Stress Questionnaire

This study employed a 21-item work stress scale developed by Frantz & Holmgren [74]. This 21-item measure is used to assess people’s work-related stress. Cronbach’s alpha is 0.93, and the scale is evaluated on a four-point Likert scale ranging from 1 “yes, always” to 5 “no, never”.

#### 3.2.3. Mental Health

The positive mental health measure was firstly developed by Lukat [75]. This scale has 9 items, and small adjustments were made to the scale statements to reflect the specific context of mental health. Cronbach’s alpha is 0.91, and all statements are evaluated on a four-point Likert scale ranging from 0 “do not agree” to 3 “agree”.

#### 3.2.4. Burnout

We also used 21-item burnout questionnaires adopted by Malach-Pines (2005) [76]. This questionnaire measures an individual’s burnout level. Seven-point Likert scale: 1 equals “never,” 2 equals “almost never,” 3 equals “rarely,” 4 equals “sometimes,” 5 equals “often,” 6 equals “very often,” and 7 equals “always”. This scale has high validity and reliability. 

#### 3.2.5. Handwashing Frequency

This study utilized the one-item measure to quantify handwashing frequency during the COVID-19 pandemic [68]. One question was, “On average, how many times did you wash or sanitize your hands every day this week?” The handwashing frequency construct has valid reliability (α = 0.91).

## 4. Results

### 4.1. Validation of Measurement Model

This study looked at the validity in terms of reliability, convergent, and discriminant using confirmatory factor analysis. Cronbach’s alpha and composite reliability (CR) values greater than 0.70 are considered as indicators of reliability [77]. Similarly, the item loading value should be larger than 0.50, and the average variance extracted (AVE) should be greater than 0.50 to establish convergent validity [78]. Table 3 shows about each construct that, all item loadings are more than 0.60, Cronbach and CR are greater than 0.70, and AVE is between 0.64 and 0.88.

As indicated in Table 3, the results reveal that this study instrument has convergent validity and reliability. Furthermore, discriminant validity is established by comparing the square root of AVE for each construct with the correlation between the construct and all others [79]. The square root of AVE exceeds the correlation between each concept and the other components in this investigation (see Table 4), indicating that all constructs have sufficient discriminant validity. Because the majority of the components in this survey were self-reported, this study looked into common technique biases (CMB). For example, to examine the CMB, we used the single-factor approach as previously published [80].

The verified data were used to test the model. The model’s overall fit index was obtained using SPSS-26 and AMOS-24. The resultant value is within the acceptable range. The root mean square error of approximation (RMSEA) was 0.032 and the standardized root mean residual (SRMR) was 0.033, both of which were less than the required threshold of 0.010 [81]. The degree of freedom (CMIN/df) was 1.28, which is, likewise, adequate. Furthermore, IFI was 0.982, TLI was 0.984, and CFI was 0.987, 0.988, all of which were greater than the acceptable 0.90 estimations.

### 4.2. Hypothesis Testing

The results of the structure model are shown in Table 4 by using the SPSS-26 version and AMOS-24 version. In particular, COVID-19 phobia has a significant negative relationship with mental health (*β* = −0.63, *p* < 0.01) and is positively related to burnout (*β* = 0.88, *p* < 0.01). Similarly, burnout also has a significant negative association with mental health (*β* = −0.53, *p* < 0.01). Furthermore, our hypotheses, such as H1, H2, and H3, are accepted.

#### 4.2.1. Mediating Effects of Burnout and Work-Related Stress

PROCESS Model 6 with 5000 bootstrap iterations was used to investigate indirect effects (mediating effect), as stated in Hypotheses 4 and 5. According to Hypothesis 4, the relationship between COVID-19 phobia and mental health will be mediated by burnout. The findings of this model are shown in Table 5 and reveal that the indirect impact of COVID-19 phobia through burnout was significant (*β* = −0.45, 95% *CI* = [0.53, 0.32]) on mental health. Similarly, it revealed that burnout significantly mediates the relationship between COVID-19 phobia and mental health, thus accepting H4. Furthermore, this model also revealed that the indirect impact of COVID-19 phobia through work-related stress on mental health was significant (*β* = −0.29, 95% CI = [0.36, 0.15]). It revealed that work-related stress significantly mediates the relationship between COVID-19 phobia and mental health, accepting H5.

#### 4.2.2. Moderating Effect of Handwashing

**Figure 2:**
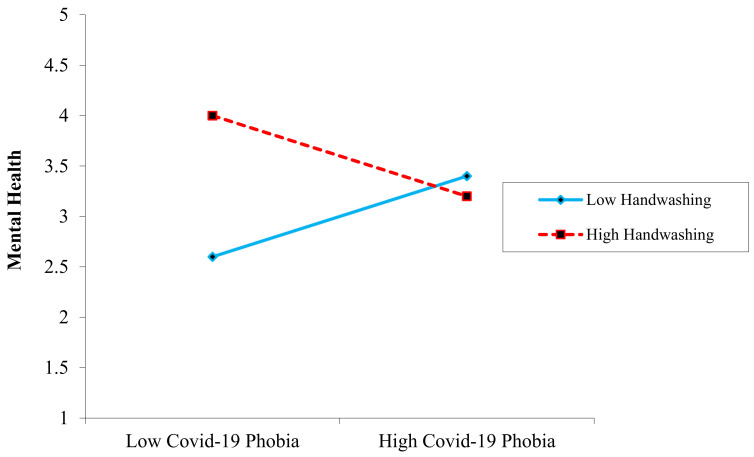
Moderating the role of handwashing on the relationship between COVID-19 phobia and mental health.

**Figure 3:**
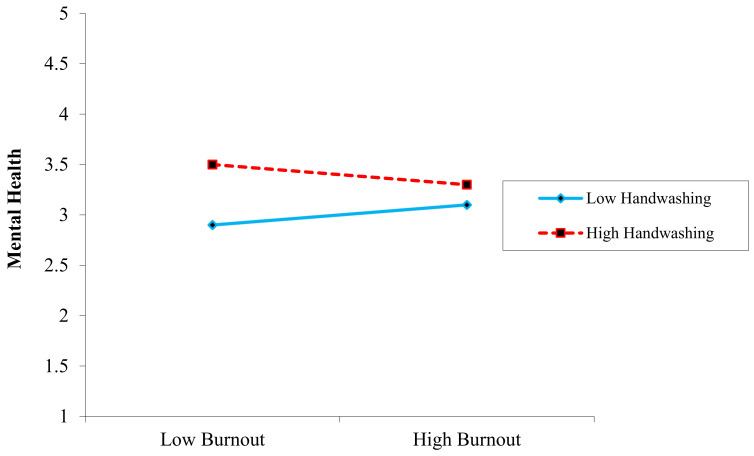
Moderating the role of handwashing on the relationship between burnout and mental health.

Hypotheses 6 and 7 predicted a moderating influence of handwashing on the relationship between COVID-19 phobia and mental health, as well as the relationship between work-related phobia and mental health. Additionally, Table 6 illustrates the moderating impact of variables. As shown in Figure 2, there was a significant interaction between COVID-19 phobia and handwashing behavior on mental health (*β* = −0.02, *SE* = 0.007, *p* < 0.01). Simple slope analyses indicated that the interaction between COVID-19 phobia and mental health was significantly negative when handwashing behavior was lower (*β* = −0.34, *t* = 5.71, *SE* = 0.053, *p* < 0.01) but not when handwashing behavior was greater (*β* = 0.06, *t* = 1.32, *p* = ns), this relationship grow into weak so H6 was accepted. Meanwhile, the interaction impact of work-related stress and handwashing behavior on mental health was non-significant (*β* = 0.032, *SE* = 0.028, *t* = −0.92, *p* = ns). Therefore, this hypothesis was rejected. The last hypothesis of our study, H8, predicted that handwashing would moderate the relationship between burnout and mental health. There was a significant interaction impact of COVID-19 phobia and handwashing behavior on mental health (*β* = −0.08, *SE* = 0.019, *p* < 0.01). Similarly, slope analyses demonstrated that the link between burnout and mental health was significantly negative when handwashing behavior was low (simple slope: *β* = 0.13, *SE* = 0.071, *t* = 3.82, *p* = 0.01), while, when handwashing behavior was higher, this relationship became weak (*β* = 0.21, *t* = 2.46, *p* = ns), as shown in Figure 3.

## 5. Discussion

In light of the growing prevalence of the omicron wave of COVID-19 and digital interaction, this study investigated the role of health care professionals’ modern interaction styles during the omicron wave. This study aims to fill the gap by examining whether current healthcare professionals suffer from mental health issues, COVID-19 phobia, work-related stress, and burnout due to the COVID-19 pandemic. The current research aimed to learn more about the mediating role of burnout and work-related stress between COVID-19 phobia and mental health and consider the moderating impact of handwashing behavior.

Our model demonstrated that COVID-19 phobia significantly negatively impacts mental health, whereas previous research has shown that phobia of illness harms mental function [82]. Videbeck and Haktanir suggested that phobia regarding objects significantly disturbs mental health. The results supported our hypotheses that COVID-19 phobia impacts burnout of healthcare professionals [83]. This study confirms the findings of studies [31,84] in which it has been theorized that phobia is closely associated with burnout. Our study confirms that phobia of contracting COVID-19 is closely linked with burnout.

This study also found that burnout has an adverse impact on the mental health of healthcare professionals. Burnout is a common occurrence in stressful situations, and it can lead to mental illness during stressful situations such as the COVID-19 pandemic [85,86]. Scholars have also proved that burnout can be caused by increasing mental health problems, such as anxiety, work stress, depression, and occupational impairment [86,87]. According to the published literature, phobia causes work distress, which is the direct source of burnout. Therefore, previous studies are in line with the current findings [88,89].

The results also show that burnout significantly mediates the relationship between COVID-19 phobia and the mental health of healthcare professionals. This finding is consistent with the view of Vignoli, who argued that burnout significantly influences the relationship between phobia and mental illness [90]. Scholars have documented that burnout is a significant cause of both phobia and mental health problems because burnout is significantly associated with high phobia and high mental problems [91]. Therefore, burnout acts as a bridge that links COVID-19 phobia to mental health problems. If burnout is ignored between COVID-19 and mental health problems, they may not be able to affect each other directly.

In addition, the results indicated that work-related stress significantly mediates the relationship between COVID-19 phobia and poor mental health. Usually, the researcher claims that work stress is a common symptom of all mental illnesses and situational phobias [92]. Furthermore, according to the theory of mind, work impairment has a significant effect on arising social phobia and mental health problems [93]. However, this study indicated that work-related stress significantly mediates the relationship between COVID-19 phobia and mental health. On the other hand, several earlier studies found that work-related stress significantly influenced mental health [94,95,96]. For example, argue that work-related stress mediates the association between COVID-19 -related phobia and mental illness among university students [97]. Therefore, our study accepted this hypothesis. This study also found support for the process model showing that the relationship between COVID-19 and mental health as well as burnout and work-related stress are each significant regarding COVID-19 phobia on mental health, as hypothesized. These findings demonstrate that handwashing behavior or attitudinal reactions differ, supporting the coping mechanism of coping theory [3]. As a result, our research discovered that handwashing behavior did not significantly moderate the relationship between work-related stress and mental health (surprisingly, rejected H7). Still, it did buffer a substantial role in the relationship between burnout and mental health (accepted H8).

### 5.1. Theoretical Contribution

From a practical aspect, our research speaks volumes about frontline health care experiences during the COVID-19 crisis, particularly in the omicron wave. We were able to capture the experiences of frontline health professionals from the cases of the first omicron wave and thus provide a fundamental understanding of how the situation has impacted healthcare workers’ lives, as well as how they might experience and deal with COVID-19 related phobia situations more broadly. To begin with, it is known that COVID-19 -related phobia has an impact on mental health, burnout, and work-related stress. It is obvious that COVID-19 phobia has consequences for work effectiveness, family engagement, and health status. Our findings also show that, in the face of COVID-19 phobia, problem-focused coping in the form of a simple behavior such as handwashing can help reduce the impact of COVID-19 -related phobia. However, we do not claim that handwashing is a universal coping mechanism that can be used in various phobia situations. Instead, we emphasize the importance of engaging in appropriate coping behaviors that are appropriate for the situation that individuals are dealing with. Handwashing has been widely recommended to combat phobia during the COVID-19 crisis. In other cases, such as when someone has a phobia or fears being laid off, taking active steps to ensure work is completed effectively may help to reduce the impact of the threat or phobia of COVID-19 and work-related stress.

In various aspects, this study added to the coping theory and mental health literature, as well as deleterious mental health variables (phobia, burnout, and work-related stress) in the context of the omicron wave. First, previous research has looked at these variables in distinct COVID-19 contexts [98,99]. In past studies, less emphasis was placed on coping behaviors such as handwashing. Such study has been requested because, in both health and organizational contexts, such research is needed to address a gap in the literature and respond to a research demand. Second, this study adds to the existing research on stress and burnout by proposing the idea of COVID-19 phobia and its link to mental health outcomes [100,101]. This study supported previous findings regarding coping theory during the omicron wave by providing empirical evidence for handwashing as a boundary condition against the negative effects of COVID-19-related phobia, burnout, and work-related stress [3]. 

The outcomes of this study demonstrated a substantial correlation between COVID-19 phobia and mental health during omicron, which is similar to an earlier study that established specific phobia negatively affects an individual’s mental health in a normal context [102,103]. 

In the H4 and H5 indirect link between COVID-19 phobia and mental health, a mediating effect of burnout usage, as well as work-related stress, were expected, and the results indicated partial mediation. This result or concept is unique in the current literature because COVID-19 phobia is a new concept in psychology. A moderating impact of handwashing was hypothesized in H6, H7, and H8, which indicated that handwashing significantly weakened the link between COVID-19 phobia and mental health, as well as COVID-19 phobia and burnout. The results confirmed these hypotheses. While H8 was rejected because COVID-19 phobia is a novel notion in psychology, this conclusion or concept is unique in the existing literature. COVID-19 phobia has yet to be researched. 

### 5.2. Practical Implications

This research has several practical implications for healthcare professionals that work with COVID-19 patients daily. This research adds to the existing body of knowledge in the following ways. To begin with, there is a rareness of well-designed studies on the links between COVID-19 phobia, burnout, work-related stress, and mental health problems in healthcare workers. We found that COVID-19 phobia and burnout in healthcare practitioners might lead to a rise in mental health issues, which can have significant psychological effects. We investigated the significant role of burnout and work-related stress in mediating the association between COVID-19 phobia and mental health problems.

A crucial requirement for causality is to investigate the directivity and mechanism between the variables. Second, we address a gap in the literature by looking at psychological variables linked to healthcare professionals’ mental health during the omicron wave of COVID-19. We expanded the previous study by involving healthcare professionals, focusing on the effects of COVID-19 on mental health. In terms of practical consequences, the research findings provide critical evidence for the development of COVID-19 phobia treatments aimed at safeguarding healthcare workers’ mental health, improving their quality of life, and making policy recommendations. It is critical to protect healthcare workers’ mental health from infection risks if they are to combat COVID-19 effectively.

Treatments for COVID-19 phobia and mental illnesses can be provided online via social networking sites, allowing for the least amount of direct contact with healthcare providers. Such interventions could aim to (a) support healthcare professionals in maintaining their mental health so that they can continue to provide primary care and health services without experiencing psychological problems during the pandemic, and (b) identify healthcare professionals who may be vulnerable to stressors due to an inability to cope with adversity during a pandemic. In addition to online therapy, psychiatric clinics can be a beneficial way to provide mental health services to healthcare workers actively fighting COVID-19 and exhibiting signs of mental health problems, including anxiety and stress-related illnesses.

The study’s findings are thought to offer insight into the nature of the secondary effects that healthcare professionals will have depending on the epidemic, as well as preventative measures to be employed for the preservation of healthcare workers’ psychological health. Given the fast development of the pandemic around the world, it is hoped that it will aid in the study of the behavioral implications of the emotional condition caused by COVID-19. Today, studies focused on the secondary effects of the outbreak are gaining traction, and comparable research concepts are likely to be developed.

Overall, based on our present research, we conclude that mental health professionals now have a crucial role in improving public wellbeing.

### 5.3. Limitations and Future Research

First, the data used to complete the proposed study were obtained from Pakistani health professionals; hence, generalization should be made only when a sample population from many nationalities and cultures has been included. This study focuses on health professionals’ relationships with COVID-19 phobia, burnout, work-related stress, and mental health, without going into depth into other elements of health professionals’ lives, such as the bio-socio model. A mediating role of burnout, as well as work-related stress, may be considered for further studies. Furthermore, the moderating role of handwashing behavior may be considered for further investigations.

In addition, the researchers propose that qualitative research utilizing narrative analysis or an interpretive phenomenological method may be used to gather real-world data to support the positivist approach used in this study. The research was only conducted in a relational and cross-sectional environment due to the pandemic’s unfavorable effects. Data were obtained physically, as well as online using the same logic and a more basic sampling technique. These should be taken into account while interpreting study results. The research only included on-the-job healthcare professionals who have not yet been affected. In this context, research involving healthcare workers who have been infected with the virus and have recovered is believed to be necessary. Furthermore, adopting multimethod or mixed methods research in terms of data diversity is thought to produce substantial results in terms of external validity. Furthermore, research focused on cross-national comparisons is believed to yield crucial findings in terms of comprehending the nature of the problem.

## 6. Conclusions

We are in the middle of a worldwide catastrophe unlike any that humanity has faced in more than a century at this point in history. COVID-19 phobia has a detrimental influence on mental health but a favorable link with burnout and work-related stress. Burnout, on the other hand, has been linked to work-related stress while having a detrimental impact on mental health. Burnout and work-related stress have been identified as critical mediators in the link between COVID-19 phobia and mental health. Additionally, handwashing behavior was found to be a major significant moderator of the relationship between COVID-19 phobia and mental health, as well as the link between burnout and mental health in healthcare professionals.

## Figures and Tables

**Table 1:** Mean and SD for all study variables for each of the 10 days.

Variables	*Day 1 M (SD)*	*Day 2 M (SD)*	*Day 3 M (SD)*	*Day 4 M (SD)*	*Day 5 M (SD)*	*Day 6 M (SD)*	*Day 7 M (SD)*	*Day 8 M (SD)*	*Day 9 M (SD)*	*Day 10 M (SD)*
C-19P	19.81 (2.11)	19.17 (2.71)	19.19 (2.69)	19.72 (2.33)	19.49 (2.43)	19.36 (2.60)	19.12 (2.86)	19.31 (2.58)	19.87 (2.11)	19.27 (2.37)
Burnout	18.23 (2.67)	18.92 (2.07)	18.57 (2.41)	18.18 (2.81)	18.84 (2.14)	18.49 (2.51)	18.58 (2.35)	18.18 (2.79)	18.57 (2.42)	18.90 (2.09)
MH	4.38 (1.61)	4.64 (1.35)	4.58 (1.39)	4.63 (1.32)	4.40 (1.57)	4.71 (1.28)	4.17 (1.81)	4.30 (1.68)	4.09 (1.92)	4.23 (1.63)
WRS	19.46 (2.51)	19.09 (2.90)	19.32 (2.67)	19.81 (2.66)	19.71 (2.29)	19.26 (2.68)	19.64 (2.38)	19.10 (2.82)	19.28 (2.68)	19.42 (2.49)
HW	1.21 (1.80)	1.61 (1.29)	0.89 (1.02)	1.88 (0.98)	1.72 (1.34)	1.41 (1.52)	0.96 (1.89)	1.18 (1.72)	1.80 (1.18)	0.88 (1.92)

Note: C-19P = COVID-19 phobia, MH = mental health, WRS = work-related stress, HW = handwashing, M = mean, SD = standard deviation.

**Table 2:** Demographics.

Variables	N	Percentage	Variables	N	Percentage
Gender			Nature of Job		
Male	41	51.9	Radiologist	18	22.8
Female	38	48.1	Pharmacist	14	17.7
Age			Cardiologist	12	15.2
Young adult	46	58.2	Oncologist	11	13.9
Middle adult	21	26.6	Nurses	24	30.4
Older adult	12	15.2	Marital status		
Job experience			Single	44	55.7
1–5 years	13	16.5	Married	35	44.3
6–10 years	28	35.4	Family status		
11–15 years	28	35.4	Higher	42	53.2
16–20 years	10	12.7	Lower	37	46.8

**Table 3:** Results of measurement analysis.

Constructs	F. L	Cronbach α	C. R	AVE
C-19P	0.842–0.939	0.89	0.87	0.71
Burnout	0.823–0.886	0.93	0.91	0.88
Mental Health	0.916–0.626	0.83	0.81	0.68
WRS	0.769–0.857	0.90	0.91	0.85
Handwashing	0.937–0.959	0.91	0.89	0.87

Note: F. L = factor loadings, C. R = composite reliability, AVE = average variance extracted. C-19P = COVID-19 phobia, WRS = work-related stress. All factor loadings are significant at the *p* < 0.001 level.

**Table 4:** Variance estimates, means, standard deviations, and intercorrelations matrix.

*Variables*	*Within-Person Variance (e^2^)*	*Between-Person Variance (r^2^)*	*% of the Within-Person Variance*	*1*	*2*	*3*	*4*	*5*
C-19P	0.61	0.38	61.81	(0.79)				
Burnout	0.84	0.97	44.71	0.88 **	(0.93)			
Mental Health	3.80	3.05	67.52	−0.63 **	−0.53 **	(0.89)		
WRS	2.97	2.99	59.83	0.96 **	0.91 **	−0.60 **	(0.91)	
Handwashing	0.39	0.62	58.92	0.32	0.82	27 **	0.19	(0.87)
M				55.82	66.26	81.71	80.02	26.02
SD				8.72	7.71	9.15	9.04	1.04

Note: * *p* < 0.05, ** *p* < 0.01, *** *p*< 0.001. *n* = 69, C-19P = COVID-19 phobia, WRS = work-related stress, M = mean, SD = standard deviation. The diagonal has information about reliability. The variables’ correlations are group-mean centered relationships among the daily variables. Estimate associations were created by aggregating variables. e^2^ (e^2^ + r^2^) was used to calculate the percentage of variance within individuals. Correlation coefficients are shown in dashed cells.

**Table 5:** Results for mediation analysis.

Direct and Indirect Effects of COVID-19 Phobia on Mental Health through Burnout and Work-Related Stress			*β*	LLCI	ULCI
C-19P	Mental Health		−0.53 ***	0.67	0.28
C-19P	Burnout	Mental Health	−0.45 **	0.53	0.32
C-19P	Mental Health		−0.40 ***	0.43	0.17
C-19P	WRS	Mental health	−0.29 **	0.36	0.15

Note: ** *p* < 0.01, *** *p*< 0.001, *n* = 69, C-19P = COVID-19 phobia, WRS = work-related stress, LLCI = lower level of confidence interval, ULCI = upper level of confidence interval.

**Table 6:** Moderating effects of handwashing on the relationship between COVID-19 phobia, burnout, WRS, and mental health.

Variables	*B*	*SE*	*t*	*p*	*R*^2^/*Sig.*
HW × C-19P on Mental health	−0.34	0.053	5.71	0.01	0.06 **
HW × WRS on Mental health	0.032	0.028	0.921	0.06	0.05 ns
HW × Burnout on Mental health	−0.13	0.071	3.82	0.01	0.08 **

Note. ** *p* < 0.01, ns = non-significant; WH = handwashing; C-19P = COVID-19 phobia WRS = work-related stress.

## Data Availability

Some or all data, models, or codes that support the findings of this study are available from the corresponding authors upon reasonable request. Informed consent was obtained from all subjects involved in the study.
